# Exosomes and Autophagy: Coordinated Mechanisms for the Maintenance of Cellular Fitness

**DOI:** 10.3389/fimmu.2014.00403

**Published:** 2014-08-20

**Authors:** Francesc Baixauli, Carlos López-Otín, Maria Mittelbrunn

**Affiliations:** ^1^Department of Vascular Biology and Inflammation, Centro Nacional de Investigaciones Cardiovasculares, Madrid, Spain; ^2^Departamento de Bioquímica y Biología Molecular, Facultad de Medicina, Instituto Universitario de Oncología, Universidad de Oviedo, Oviedo, Spain

**Keywords:** exosomes, autophagy, proteostasis, spreading, endosome, multivesicular bodies, lysosome, neurodegeneration, aging

## Abstract

Conditions resulting from loss of cellular homeostasis, including oxidative stress, inflammation, protein aggregation, endoplasmic reticulum stress, metabolic stress, and perturbation of mitochondrial function, are common to many pathological disorders and contribute to aging. Cells face these stress situations by engaging quality control mechanisms aimed to restore cellular homeostasis and preserve cell viability. Among them, the autophagy-lysosomal pathway mediates the specific degradation of damaged proteins and organelles, and its proper function is related to cellular protection and increased life span in many model organisms. Besides autophagy, increasing evidence underscores a role for exosomes in the selective secretion of harmful/damaged proteins and RNAs and thus in the maintenance of cellular fitness. In this perspective article, we discuss the emerging function of exosomes as a means of alleviating intracellular stress conditions, and how secretion of harmful or unwanted material in exosomes, in coordination with the autophagy-lysosomal pathway, is essential to preserve intracellular protein and RNA homeostasis. Finally, we provide an overview about the consequences of the spreading of the exosome content in physiological and pathological situations, and suggest putative therapeutic strategies for these exosome-mediated alterations.

## Introduction

The degradation and recycling of cellular material is essential for eukaryotic cells to maintain their homeostasis. Extracellular material and plasma membrane proteins are delivered to lysosomes for degradation via the endocytic pathway, whereas cytosolic components and organelles are delivered to the lysosomes by autophagy. After a complex maturation process, autophagosomes and endosomes converge in the lysosome to deliver their cargo for degradation. Autophagy is used by all cell types to overcome starvation, recycle nutrients, and remove unwanted or damaged intracellular constituents, including both proteins and organelles. In some situations, cells remove this unwanted or damaged material through their release to the extracellular environment as exosomes. Since exosomes can be transferred from one cell to another, secretion of unwanted material to the extracellular environment in exosomes may have an impact, which can be beneficial or detrimental, in neighboring cells.

In this perspective article, we discuss the molecular and functional crosstalk between exosome release and autophagy pathways. We also discuss how conditions regulating the metabolic state of the cell or challenging stress conditions may affect both processes and contribute to the maintenance of intracellular homeostasis. Finally, we discuss how exosome release may serve as a cellular mechanism to partially bypass the autophagic defect that occurs during aging or in diverse pathological situations.

## Exosome Secretion and Autophagy are Coordinated Mechanisms

*Exosomes* are small vesicles that are released by almost every cell type to the extracellular environment. Contrary to other types of extracellular vesicles, exosomes have endocytic origin and are formed as intraluminal vesicles (ILVs) by inward budding of the limiting membrane of late endosomes or multivesicular bodies (MVBs) (1). Exosome secretion occurs in a constitutive manner although cellular stress or activation signals modulate their secretion (2). Exosomes carry specific repertoires of proteins and nucleic acids in the form of mRNAs and small non-coding RNAs, including microRNAs, and are considered as an unconventional secretory pathway. Exosomes can transfer their content to neighboring cells and regulate at a distance the properties of receptor cells (3). Consequently, exosomes have been found to play a role in intercellular communication in several physiological processes, and contribute to organism development (4), immune responses (5), neuronal communication (6), and tissue repair (7). However, exosomes may participate in some pathological disorders, favoring tumor progression (8) or virus spreading (9). Additionally, given that exosomes carry damaged cellular material targeted for destruction, they facilitate the spreading of toxic forms of aggregated proteins such as α-synuclein, β-amyloid, and prion proteins and thus contribute to the progression of neurodegenerative diseases (10).

Loading of proteins into exosomes is controlled through a variety of pathways, most of which are still not fully understood (11). The endosomal sorting complexes required for transport (ESCRT) machinery is essential for the sorting of ubiquitinated membrane proteins and for the formation of ILVs in the MVB compartment. ESCRT is composed of four multimeric complexes, ESCRT-0 to III, and the VPS4 ATPase that mediates the final ESCRT disassembly and budding of the ILVs. ILVs budding and protein sorting depend also on tetraspanin and lipid-dependent interactions (2). An active sorting mechanism participates in RNA targeting into exosomes, which allows some RNA species to be particularly enriched in exosomes, whereas other RNAs are barely detected (12). Next-generation sequencing analysis of exosomal RNA revealed that the most abundant RNA species are small ribosomal RNA (rRNA), fragmented tRNAs, and structural RNAs (13). Notably, exosomes also contain certain microRNAs and mRNAs. Regarding microRNA composition, a specific repertoire of microRNAs is found in exosomes (12–15). The loading of microRNA into exosomes depends on a tetranucleotide sequence recognized by heterogeneous nuclear ribonucleoproteins (hnRNPs) (12). Once the ILVs are formed, MVBs can fuse with the plasma membrane and release their content to the extracellular environment as exosomes. Alternatively, MVBs fuse with lysosomes where the content of the ILVs is degraded. Although a great deal of effort has been placed on understanding the mechanisms of exosome cargo loading, less is known about the signals and the metabolic clues that coordinate the fate of MVBs between exosome secretion or their integration with the degradative and recycling pathways of the cell.

Autophagy is a degradative pathway critical in the maintenance of protein homeostasis (proteostasis) as well as the preservation of proper organelle function by selective removal of damaged organelles. Autophagy occurs constitutively but can also be induced in response to cellular stresses including limitations to various types of nutrients, such as amino acids, growth factors, oxygen, and energy, excessive ROS or DNA damage (16). Autophagy represents an essential cytoprotective pathway that participates in the maintenance of cellular fitness by several mechanisms. Autophagy may act as a proteoquality control mechanism that continuously degrades pre-existing cellular material and provides building blocks for the renewal of cellular components. Degradation of self-components by autophagy is a critical survival response against starvation conditions, as it enables recycling of macromolecules to provide new nutrients and energy. Moreover, autophagy leads to the elimination of potentially toxic aggregates and limits the accumulation of ubiquitinated proteins. Autophagy is also a critical regulator of organelle homeostasis, particularly of mitochondria (17). Autophagy allows the selective removal of dysfunctional mitochondria, which release pro-apoptotic factors and generate oxygen species. To date, three autophagy-related pathways have been described, which promote bulk as well as selective degradation of cytosolic and organelle components. In macroautophagy (herein autophagy), whole cytosolic regions are sequestered inside double-membraned vesicles (autophagosomes) that are then able to fuse either with endocytic vesicles (as MVB) or lysosomes, which provide the hydrolytic enzymes that will degrade autophagosomal content (18). The chaperone-mediated autophagy (CMA) is a more selective autophagy pathway that relies on the chaperone hsc70, which specifically recognizes cytosolic substrate proteins that contain KFERQ-like pentapeptide motifs. The transmembrane protein LAMP-2A acts as a receptor for hsc70 on the lysosome to deliver unfolded proteins into the lysosomal lumen for degradation in a type of autophagy that does not require membrane reorganization (19). The third type of autophagy, microautophagy, involves engulfment of small cytoplasmic components by inward invagination of the lysosomal membrane (20).

**Figure 1:**
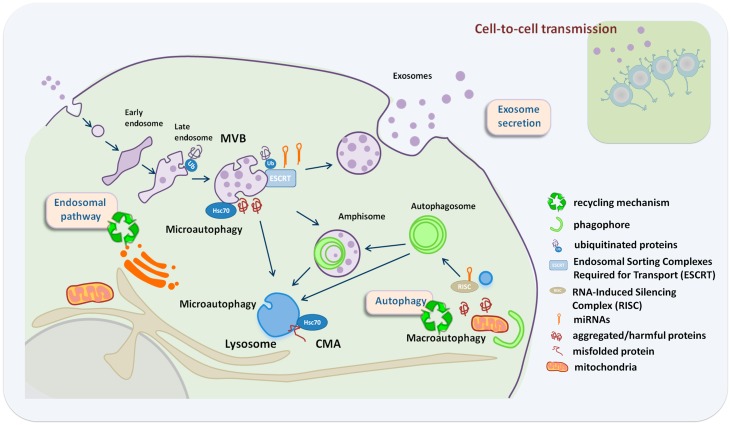
**Crosstalk between exosome secretion and autophagy in maintenance of cellular homeostasis**. Exosome secretion is an alternative way to alleviate stress when recycling pathways are compromised, with an impact in neighboring cells. Recycling mechanisms relay on endosome/autophagy and lysosomal function. Recycling pathways include: macroautophagy where whole cytosolic regions are sequestered inside autophagosomes that fuse either with MVBs or lysosomes, chaperone-mediated autophagy (CMA) where LAMP-2A acts as a receptor for hsc70 on the lysosome to deliver unfolded proteins into the lysosomal lumen, and microautophagy that involves engulfment of small cytoplasmic components by inward invagination of the lysosomal or endosomal membrane.

Several lines of evidence point to a close relationship between the different autophagy pathways and the biogenesis and secretion of exosomes (Figure 1). The mechanisms that guide the selective incorporation of proteins during exosome biogenesis and the membrane invagination that occurs during ILV formation and MVB maturation have been proposed to be a type of endosomal microautophagy. This endosomal microautophagy transports cytosolic proteins into ILVs relying on the ESCRT machinery and the chaperone hsc70. The process does not require substrate unfolding or the essential component of CMA in lysosomes, LAMP-2A, but it relies on electrostatic binding of hsc70 to endosomal acidic phospholipids (21).

During macroautophagy, autophagosomes can either fuse with lysosomes or with MVBs. Studies on ESCRT mutants have revealed a close relationship between the process of autophagy and MVB biogenesis. ESCRT mutants are unable to complete autophagic maturation due to the lack of autophagosome fusion with the endolysosomal system (22–24) and exhibit increased number of autophagosomes. In different *C. elegans* ESCRT mutants, autophagic activity is increased, which allows cells to deal with the detrimental accumulation of abnormal endosomes, resulting from defects in the ESCRT machinery. In this context, autophagy induction contributes to increase cell survival and organismal lifespan presumably through the selective removal of late endosomes by the autophagy machinery (25). Remarkably, autophagy modulators regulate MVB formation and the exosome release (26). Autophagy induction by starvation, rapamycin treatment, or LC3 overexpression inhibits exosome release, suggesting that under conditions that stimulate autophagy, MVBs are directed to the autophagic pathway with consequent inhibition of exosome release (27). Hence, the balance between autophagy induction and exosome release might be regulated by the cellular metabolic state. The challenge now is to understand how metabolic sensors regulate the fate of certain molecules toward autophagic degradation or secretion in exosomes, and how this regulation impacts the autonomous and non-cell autonomous homeostasis.

Selective autophagy as well as MVBs participates in the regulation of cellular RNA homeostasis. Assembly of the silencing machinery to intracellular endomembranes, specifically to late endosomes and MVBs, is critical in the regulation of miRNA activity and might determine the fate of certain miRNAs to be exported in exosomes. Consistently, mutations in ESCRT components lead to a decrease in miRNA activity (28, 29). Moreover, autophagy is also involved in the regulation of miRNA activity by promoting selective degradation of DICER and AGO2 (30). Interestingly, the lysosomal membrane protein LAMP-2A interacts with RNA-binding proteins, such as hnRNPs, nucleophosmin, or ribosomal proteins, and has been suggested to mediate the import and degradation of RNA molecules into lysosomes (31). The sorting of specific set of miRNAs into exosomes depends on hnRNPs (12). Whether LAMP-2A, nucleophosmin, and other RNA-binding proteins are essential for the import of RNA molecules into the endolysosomal system and their export in exosomes needs further investigation.

Finally, another line of evidence supports the role of autophagy and exosomes in mediating unconventional secretion for proteins that lack a typical signal peptide (32). This includes a role for autophagy and MVBs in regulating unconventional secretion of ACB1 in yeast (33, 34) and interleukin-1β in mammalian cells (35, 36).

## Autophagy Dysregulation and Exosome Secretion in Human Diseases

Autophagy malfunction is often linked to a variety of human diseases, such as cancer, neurodegeneration, and microbial infection, among many others (37). Neurodegenerative diseases are an example to illustrate how autophagy dysfunction is linked to exosome release. Autophagy dysregulation in neurons may increase the secretion and the intercellular transmission of toxic proteins in exosomes and thus, contribute to the spreading of the neurodegenerative diseases (38).

In this regard, it is well established that loss of basal autophagy causes *neurodegeneration* (39, 40). The role of autophagy as a degradative pathway is critical since it prevents aggregation of proteins, such as hungtintin, tau, and alpha-synuclein, which are associated with neurodegenerative diseases (41, 42). Impairment of autophagy is accompanied by accumulation of p62 (sequestosome 1/SQSTM1), which leads to the formation of large protein aggregates containing both p62 and polyubiquitinated proteins (43). Similar inclusion bodies with p62 and ubiquitin have been identified in various neurodegenerative pathologies, including Alzheimer disease, Parkinson disease, and amyotrophic lateral sclerosis (ALS). Pharmacological activation of autophagy can mitigate the accumulation of aggregated pathological proteins and cell death indicating that autophagy can attenuate proteotoxicity (44). Interestingly, MVBs and the ESCRT machinery have also been involved in neurodegenerative diseases. In fact, it has been reported that ESCRT-III dysfunction causes autophagosome accumulation and neurodegeneration (23). Moreover, depletion of ESCRT subunits (Tsg101, Vps24) or overexpression of a *CHMP2B* mutant associated with frontotemporal dementia, inhibits autophagic degradation, and leads to accumulation of ubiquitin-positive aggregates containing aggregate-prone proteins associated with neurodegenerative diseases (22, 23). These data indicate that functional MVBs are required to prevent accumulation of abnormal proteins that can disrupt neural function and lead to neurodegeneration. However, functional MVBs might be also involved in the loading and secretion of misfolded and harmful proteins in exosomes with deleterious effect on neighboring cells (45). Exosomes are involved in the spread of toxic proteins in neurodegenerative diseases such as Alzheimer, Huntington, Parkinson, and prion diseases (46). In addition, misfolded proteins involved in ALS, such as SOD, TDP-43, and hnRNPA2B1, have been found in exosomes (12, 47–49). Upon stress, TDP-43, hnRNPA2B1, and FUS proteins exit the nucleus and accumulate in stress granules, whose clearance depends on autophagy and valosin-containing protein (VCP) function (50). Mutations in VCP predispose humans to ALS, frontotemporal lobar degeneration, and multisystem proteinopathy, suggesting that autophagic clearance of stress granules may be important in the context of neurodegenerative diseases. Thus, it is possible that protein aggregates, which are not properly cleared by autophagy, may spread to neighbor neurons in a prion-like manner. In this regard, there is accumulating evidence that extracellular protein aggregates can be taken up by cells (38), thus promoting the formation of new protein aggregates in the recipient cell. Recent studies on alpha-synuclein (αsyn) oligomers also support our proposal of links between exosomes and autophagy. These oligomers can be secreted either directly or associated with exosomes, being the secretion route strongly influenced by autophagic activity (51). These findings suggest that exosome-mediated release of αsyn oligomers is a mechanism whereby cells clear toxic αsyn oligomers when authophagic mechanisms fail. Accordingly, we propose that prevention of exosomal release by promoting autophagy might be a novel approach to develop new drugs for treatment of neurodegenerative diseases.

On the other hand, it is also well established that during aging, there is a decline in overall proteolytic activity and progressive intracellular accumulation of damaged macromolecules and organelles (52). Insufficient digestion of damaged molecules, which lead to a progressive accumulation of deleterious material, might promote the release of partially digested or undigested materials through exosomes. This is important, for example, during age-related macular degeneration (AMD), where increased autophagy and the release of exosomes may contribute to the formation of drusen (53). Autophagy appears to decline with age, and several key players in the autophagic pathway (ATG5 and ATG7) show decreased expression in the brains of aging individuals (54). In addition, a decrease in LAMP-2A is the responsible for diminished CMA activity during aging (55). Interestingly, senescence causes an increase in exosome secretion, which is dependent on the activation of the p53 tumor suppressor (56, 57). However, how autophagy and exosome secretion are coordinated during organismal aging is still a largely unexplored question. Further studies will be necessary to evaluate whether exosomes have the ability to transmit systemic signals, which can contribute to explain the synchrony characteristic of the aging process.

## Final Remarks

The selective removal and secretion of harmful proteins in exosomes or by the autophagy-lysosomal pathway are coordinated processes that participate in protein homeostasis and contribute to the maintenance of cellular fitness. Autophagy is a protective process due to its ability to remove noxious cellular components and also because it provides energy and basic cellular building blocks during adverse conditions, as nutrient deprivation. In parallel, exosome secretion may be involved in the spreading of signals to surrounding cells to coordinate systemic responses. Thus, we propose that exosome secretion may function in close relation with autophagy pathway to preserve protein and RNA homeostasis, and to mediate the spreading of signals to surrounding cells in order to coordinate organismal systemic responses. Understanding the apparently intricate liaisons between autophagic regulation and exosome secretion constitutes an interesting challenge, now that both manipulation of autophagy and exosome secretion is being considered as a therapeutic strategy. Hopefully, these studies may lead to the development of novel approaches for therapeutic intervention in diseases associated with alterations of proteostasis.

## Conflict of Interest Statement

The authors declare that the research was conducted in the absence of any commercial or financial relationships that could be construed as a potential conflict of interest.
